# The spatial distribution of radiodense breast tissue: a longitudinal study

**DOI:** 10.1186/bcr2318

**Published:** 2009-06-03

**Authors:** Snehal M Pinto Pereira, Valerie A McCormack, Sue M Moss, Isabel dos Santos Silva

**Affiliations:** 1 Cancer Research UK Epidemiology and Genetics Group, Department of Epidemiology and Population Health, London School of Hygiene and Tropical Medicine, Keppel Street, London WC1E 7HT, UK; 2 Current address: International Agency for Cancer Research, 150 Cours Albert Thomas, Lyon 69008, France; 3 Cancer Screening Evaluation Unit, The Institute of Cancer Research, Sutton SM2 5NG, UK

## Abstract

**Introduction:**

Mammographic breast density is one of the strongest known markers of susceptibility to breast cancer. To date research into density has relied on a single measure (for example, percent density (PD)) summarising the average level of density for the whole breast, with no consideration of how the radiodense tissue may be distributed. This study aims to investigate the spatial distribution of density within the breast using 493 mammographic images from a sample of 165 premenopausal women (~3 medio-lateral oblique views per woman).

**Methods:**

Each breast image was divided into 48 regions and the PD for the whole breast (overall PD) and for each one of its regions (regional PD) was estimated. The spatial autocorrelation (Moran's *I *value) of regional PD for each image was calculated to investigate spatial clustering of density, whether the degree of clustering varied between a woman's two breasts and whether it was affected by age and other known density correlates.

**Results:**

The median Moran's *I *value for 165 women was 0.31 (interquartile range: 0.26, 0.37), indicating a clustered pattern. High-density areas tended to cluster in the central regions of the breast, regardless of the level of overall PD, but with considerable between-woman variability in regional PD. The degree of clustering was similar between a woman's two breasts (mean within-woman difference in Moran's *I *values between left and right breasts = 0.00 (95% confidence interval (CI) = -0.01, 0.01); *P *= 0.76) and did not change with aging (mean within-woman difference in *I *values between screens taken on average 8 years apart = 0.01 (95% CI = -0.01, 0.02); *P *= 0.30). Neither parity nor age at first birth affected the level of spatial autocorrelation of density, but increasing body mass index (BMI) was associated with a decrease in the degree of spatial clustering.

**Conclusions:**

This study is the first to demonstrate that the distribution of radiodense tissue within the breast is spatially autocorrelated, generally with the high-density areas clustering in the central regions of the breast. The degree of clustering was similar within a woman's two breasts and between women, and was little affected by age or reproductive factors although it declined with increasing BMI.

## Introduction

Mammographic breast density is one of the strongest known markers of susceptibility to breast cancer. A recent meta-analysis of incident studies found that women with very dense breasts (that is, radiologically dense tissue occupying > 75% of the gland) have a fourfold to sixfold increased risk of developing breast cancer relative to women with little density (that is, < 5% dense tissue) [1]. It has been estimated that breast density in 50% or more of the breast may account for about one-third of all breast cancer cases in developed populations [2,3].

Most research into breast density has relied on a single measure (for example, percent density (PD)) to summarise the average level of density across the whole breast, with no consideration of how the radiodense tissue may be distributed. Knowledge of the spatial distribution of the dense tissue, and how it may change with age and other density correlates, however, may improve our understanding of the pathogenesis of breast cancer. This knowledge could help to clarify whether breast density is a general marker of susceptibility to breast cancer or a more specific and localised marker of risk, with cancers arising within the densest areas of the breast [4,5]. It is also conceivable that the location of radiodense tissue may affect breast cancer risk, independently of the average level of density for the whole breast, in the same way as cancers arising in different locations of the breast appear to have different characteristics and prognostic outcomes [6].

It is well established that average density across the whole breast tends to decrease with age [7,8], with some of the largest decreases occurring with the menopause [7], and within a woman density varies slightly between left and right breasts [9]. Whether the distribution of density changes with age, however, is unclear; that is, whether the rate of decline in density is similar across the whole breast or whether it varies according to the region of the breast. To our knowledge no previous study has examined the distribution of breast density, and its variability within and between women. Moré et *al*. commented that the highest density point was often found in the central breast region, but examination of the density distribution was not the aim of their study [10].

The aim of the present study is to investigate the spatial distribution of density within the breast. The specific study objectives are to examine the distribution of density across regions of the breast, to investigate whether spatial distribution of density is similar in a woman's left and right breasts, to assess whether the spatial distribution of density changes with age and other known correlates of density, and to determine how the location of the point of highest density in a breast relates to its regional density distribution.

## Materials and methods

### Study population

Participants were sampled from a previous study [11]. Briefly, the Mammography, Oestrogens and Growth Factors study is an observational study nested within the Age Trial, a trial of annual mammographic screening conducted in Britain [12]. Women randomised to the intervention arm (approximately 54,000) between 1991 and 1997 were offered annual breast screening from age 39 to 41 years up to and including the calendar year of their 48th birthday. Screening in the trial was by two-view mammography – cranio-caudal and medio-lateral oblique (MLO) views – at the first screen and mostly by single MLO view thereafter. Between 2000 and 2003, women in the intervention arm who were still participating in the trial were invited to join the Mammography, Oestrogens and Growth Factors study by providing a blood sample and completing a questionnaire. Over 8,000 women enrolled. For a substudy on endogenous hormones and density [11], all 800 Caucasian women from the Mammography, Oestrogens and Growth Factors study who were cancer free, who were still having regular menstrual cycles and who were not on hormone replacement therapy or oral contraceptives were invited to participate by providing urine samples throughout the menstrual cycle. A total of 533 women provided repeat urine samples. Mammograms were available for 494 of these women.

In the present paper, the woman's screen closest to her urine sample is referred to as her exit screen, and the screen taken at entry into the Age Trial as her entry screen. Only the exit and entry screens were included in this analysis.

These 494 women were classified into five groups (< 20%, 20 to 39%, 40 to 59%, 60 to 79% and ≥ 80%) on the basis of their PD values at the exit screen from the left MLO film (see Mammographic measurements for details). Approximately 40 women from each group were randomly chosen to be included in the present study except for the highest-density group, in which there were only five women and therefore all of them were selected.

The study was approved by the South East Research Ethics Committee (01/1/46) and all participants provided written informed consent.

### Mammographic measurements

All mammograms for the 494 women were digitised using an Array 2905 laser digitiser with optical density range 0 to 4.0, 12-bit depth, and a pixel size of 50 μm (Array Corporation Europe, Roden, the Netherlands). Density readings were performed by a single reader (IdSS) using the interactive threshold method as implemented by the Cumulus software [13]. This method dichotomises pixels on a digitised mammogram according to their intensities, into dense and nondense using the threshold defined by the user. The Cumulus software selects all areas at least as dense as the defined threshold and automatically calculates the total area of dense tissue and the total area of the breast as well as their ratio (PD, expressed as a percentage). The images were read blindly in a random order and with high reliability (intraclass correlation coefficient for the PD measurements = 0.91; 95% confidence interval (CI) = 0.87, 0.94).

**Figure 1:**
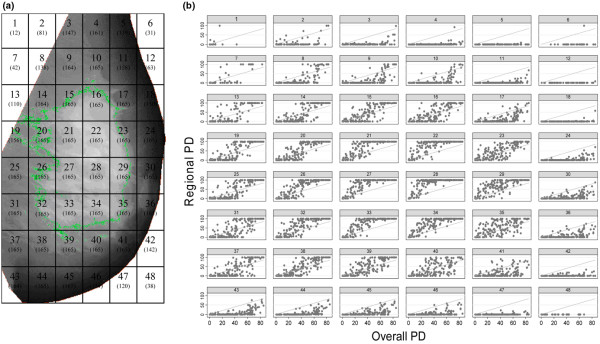
Plots of regional versus overall percent density for all study participants. **(a) **Schematic representation of the defined 48 regions in the breast (numbers in brackets indicate number of women who had breast tissue in the relevant region at the left exit medio-lateral oblique (MLO) screen). **(b)** Regional percent density (PD) plotted against overall PD (at the left exit MLO mammograms), by region.

As the MLO view was available at both entry and exit screens, this view was used in the present study. Each MLO image was automatically partitioned into 48 rectangular regions (a grid of six columns and eight rows), by dividing the distance from the chest wall to the nipple into six equally-sized segments, and the distance from the top of the image to the bottom into eight segments (Figure 1a). These regions were created by taking the coordinates of all the points used to delimit the breast area in the Cumulus software (saved in the Masking table) – that is, around the pectoral and breast edges – and importing them into Stata (Stata Corp., College Station, TX, USA). From these data we developed a program to identify, for each image, the top and bottom of the breast (that is, the minimum and maximum *y *coordinates) and the chest wall and nipple (that is, the minimum and maximum *x *coordinates). The *y *and *x *ranges were divided by eight and six, respectively, to generate four coordinates to define the rectangles corresponding to each one of the 48 regions. The coordinates of the four points delimiting a given region were then reincorporated into the Cumulus software, where they were used to automatically mask everything outside that particular region.

The size of the regions was image specific because of varying breast sizes, but the relative position of regions across images was similar. Owing to different breast positioning, some regions did not always correspond to the breast; for example, regions 1, 2, 7, 8 and 13 (Figure 1a) might correspond to the pectoral muscle in some images. Regions around the breast edge did not fill the full rectangular area, but these regions were still included and the PD calculated from the reduced area. The numbers of women contributing to each region, at the left exit MLO mammogram, are indicated in Figure 1a; regions for which no women contributed to subanalyses of the data are marked X in the figures.

Once the PD of the whole image (overall PD) was read using the Cumulus software, the PD in each one of the 48 regions (regional PD) was estimated by electronically masking the remaining regions of the breast and using the same density threshold defined when reading the corresponding image-specific overall PD for the whole breast.

### Statistical methods

The distribution of regional density was examined cross-sectionally in the left exit MLOs by plotting, for each one of the 48 regions, the regional PD values for the 165 participants against their overall PDs. Mean regional PD values, as estimated by the average across all participants, and standard deviations (SDs) of regional PDs were also displayed according to the predefined overall PD groups.

Moran's *I *statistics [14] were estimated to evaluate spatial autocorrelation in regional PDs. Moran's *I *values range from (approximately) -1 to 1, with positive values indicating positive autocorrelation (that is, a clustered pattern in which adjacent regional PDs are more correlated than those further apart), negative values indicating negative autocorrelation (that is, a dispersed pattern), and a zero value indicating no autocorrelation (that is, a random pattern). A weight matrix is required in the calculation of Moran's *I *values, so that regions close to each other are given a greater weight than those located further apart; the weights were defined as 1/*d*^2^, where *d *represents the straight line distance between the mid-points of two regions.

To assess determinants of the degree of spatial autocorrelation, Moran's *I *values were regressed against overall PD, body mass index (BMI), parity and age at first birth (among parous women). Paired *t *tests of the within-woman difference in Moran's *I *values between the left and right breasts, and between the entry and exit left MLO screens, were performed to establish whether the degree of spatial autocorrelation differed between breast sides or changed with aging. To assess whether the location of low and high PD clusters varied between women, Moran's *I *values were also calculated on a single, averaged image representing mean regional PDs for all 165 participants (or all those in a given overall PD stratum) combined.

Finally, we investigated how the point of highest density within a woman was related to her regional PD. The point of highest density (that is, the point with maximum pixel intensity) was identified using the highest possible density threshold to identify the smallest possible dense area (excluding calcifications and skin folds) and its centre taken as the point of highest density. If this procedure identified more than one area of highest density, these areas were delimited and the centre of each of them taken as points of highest density. To assess within each woman whether the location of the highest density point changed between her entry and exit left MLO screens, the region where this point was located on each screen was identified and the distance between their mid-points measured in terms of the smallest number of regions they were apart. Within-woman clustering of these distances was accounted for using a random-intercept multilevel model. To establish whether the relative difference between the overall threshold and the threshold corresponding to the point of highest density was consistent across images, the standardised proportional increase in threshold value was calculated:



Analyses were conducted in Stata version 10 software (Stata Corp., College Station, TX, USA), and the figures were produced in Stata and ArcGIS 9.2 software (ESRI, Redlands, California, USA).

## Results

A total of 165 Caucasian women were randomly chosen, stratifying by PD of the left exit MLO mammogram, and all but one participant had an entry mammogram (this woman attended only one screening round). The time-lag between the entry and exit screens ranged from 4.0 to 12.2 years (mean (SD) = 8.1 (1.6) years). Self-reported BMI information was available for 162 women and this was acquired on average within 1 year of the exit screen (mean (SD) = 1.1 (1.2) years). The mean BMI decreased markedly with increasing PD, whereas nulliparity and mean age at first birth (among parous women) tended to increase. The mean dense area was higher and the mean lucent area was much lower with increasing PD for both the entry and exit screens (Table 1).

**Table 1:** Characteristics of the study subjects and their mammographic features

		Percent density group at left exit mammogram					
		
	Number of women	< 20% (n = 41)	20 to 39% (n = 43)	40 to 59% (n = 38)^a^	60 to 79% (n = 38)^b^	≥ 80% (n = 5)	All women
Characteristic							
Entry screen age (years)	164	40.6 (0.8)	40.6 (0.8)	40.7 (1.0)	40.4 (0.8)	41.4 (1.1)	40.6 (0.9)
Exit screen age (years)	165	48.6 (1.3)	48.6 (1.5)	48.4 (2.1)	48.9 (1.8)	48.5 (1.7)	48.6 (1.7)
Height (m)	164	1.6 (0.1)	1.6 (0.1)	1.6 (0.1)	1.6 (0.1)	1.7 (0.1)	1.6 (0.1)
Weight (kg)	163	77.3 (13.7)	67.1 (11.9)	63.0 (12.6)	58.8 (7.6)	54.2 (8.2)	66.4 (13.6)
Body mass index (kg/m^2^)	162	29.1 (4.9)	25.5 (4.1)	23.7 (4.1)	22.0 (2.6)	19.9 (1.8)	25.1 (4.8)
Age at first birth (years)	138	24.8 (5.1)	25.3 (5.1)	27.2 (4.9)	27.4 (4.3)	27.7 (7.8)	26.1 (5.0)
Number of births							
0	27 (16.4)	6 (14.6)	4 (9.3)	6 (15.8)	9 (23.7)	2 (40.0)	
1	18 (10.9)	4 (9.8)	7 (16.3)	4 (10.5)	1 (2.6)	2 (40.0)	
2	79 (47.9)	19 (46.3)	22 (51.2)	19 (50.0)	18 (47.4)	1 (10.0)	
3+	41 (24.9)	12 (29.3)	10 (23.3)	9 (23.7)	10 (26.3)	0 (0.0)	
Mammographic features							
Entry screen, left breast							
Total breast area (cm^2^)	164	168.8 (70.0)	123.6 (32.9)	111.8 (43.7)	100.2 (29.4)	106.4 (48.3)	126.3 (53.2)
Dense area (cm^2^)	164	16.3 (13.0)	38.8 (19.4)	52.1 (22.4)	61.7 (20.9)	80.1 (32.2)	42.8 (26.5)
Lucent area (cm^2^)	164	152.5 (76.8)	84.8 (33.9)	59.6 (36.2)	38.5 (20.7)	26.3 (17.3)	83.5 (63.6)
Percent density (%)	164	13.0 (13.4)	32.6 (15.5)	48.0 (15.4)	62.5 (13.3)	76.9 (5.6)	39.5 (23.9)
Exit screen, left breast							
Total breast area (cm^2^)	165	194.0 (76.3)	144.6 (44.1)	119.1 (40.6)	105.6 (30.9)	102.1 (37.0)	140.7 (60.8)
Dense area (cm^2^)	165	16.6 (10.3)	42.3 (14.3)	55.2 (17.7)	69.8 (19.7)	83.7 (28.9)	46.5 (25.9)
Lucent area (cm^2^)	165	177.5 (77.1)	102.3 (34.1)	63.9 (25.0)	35.8 (12.6)	18.4 (8.2)	94.3 (69.7)
Percent density (%)	165	9.6 (6.1)	29.7 (5.5)	47.0 (5.8)	66.4 (4.4)	82.5 (1.8)	38.7 (22.7)

Data presented as mean (standard deviation) or *n *(%). ^a^There were 37 women at entry and 38 women at exit; weight and body mass index are missing for one woman in this group. ^b^Height and weight missing for one woman (each); body mass index missing for two women in this group.

Among women with high overall PD, the regional PD in the centre of the breast (that is, regions 13 to 16, 19 to 23, 25 to 29, 31 to 35 and 37 to 40; Figure 1) was close to 100%. Other regions (that is, regions 4 to 6, 11, 12, 18, 24, 42, 43, 47 and 48) had very low PD, regardless of the value of the overall PD, and they tended to be located along the skin edge (Figure 1b). If breast density was evenly distributed across the breast and had no spatial pattern, the regional PD values would have been close to and randomly distributed about the overall PD (that is, the points would be randomly scattered on either side of the unity lines in Figure 1b). This was not the case, indicating that there was a spatial pattern to the distribution of density within the breast.

**Figure 2:**
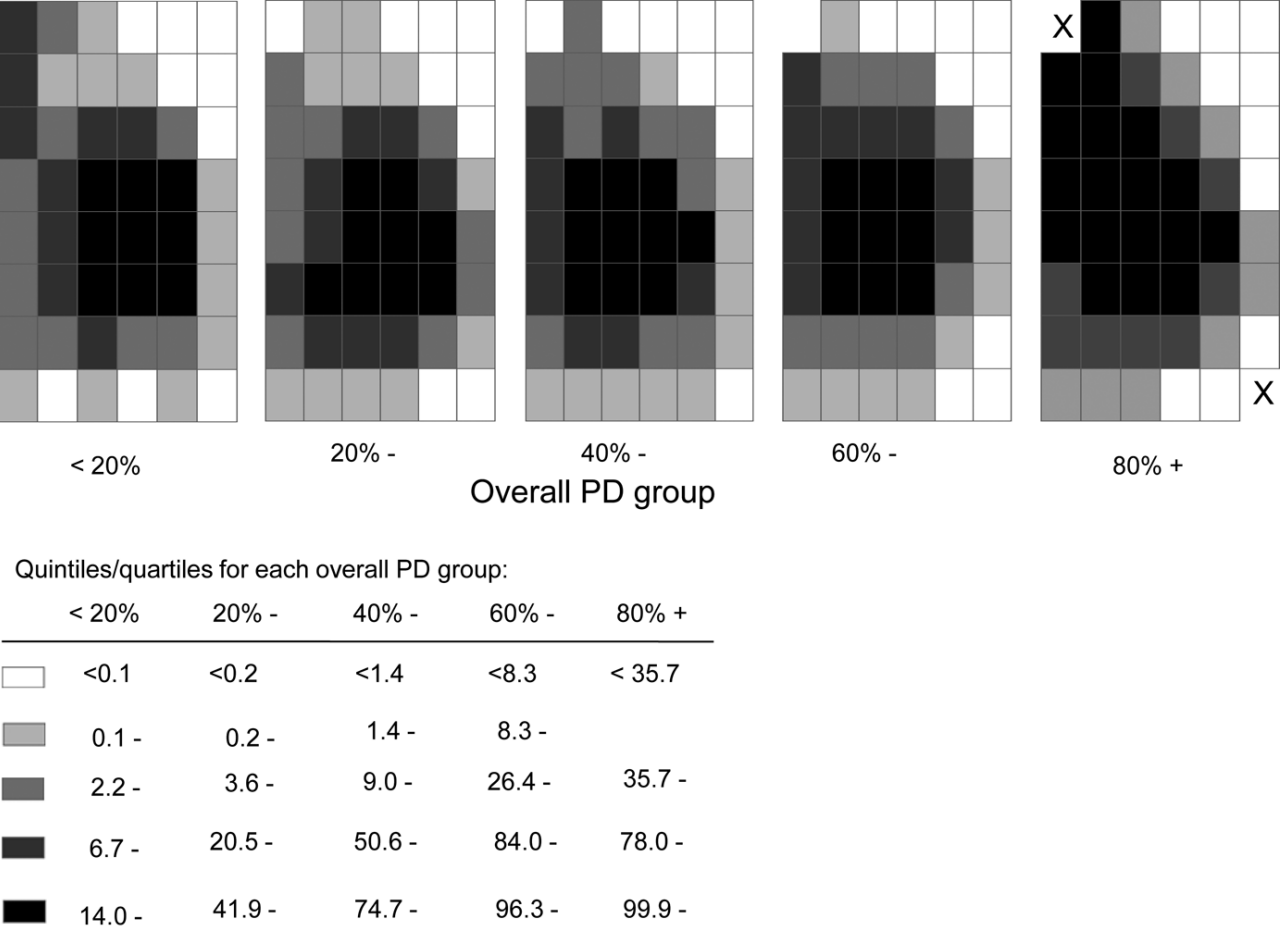
Distribution of mean regional percent density, by level of overall percent density. Distribution of mean regional percent density (PD), as estimated by the average across participants in each overall PD group, at the left exit medio-lateral oblique mammograms. Group-specific quintiles were used to categorise the mean regional PD except in the highest overall PD group, where quartiles were used because of the small number of participants. X, region where no women contributed to that particular analysis.

**Figure 3:**
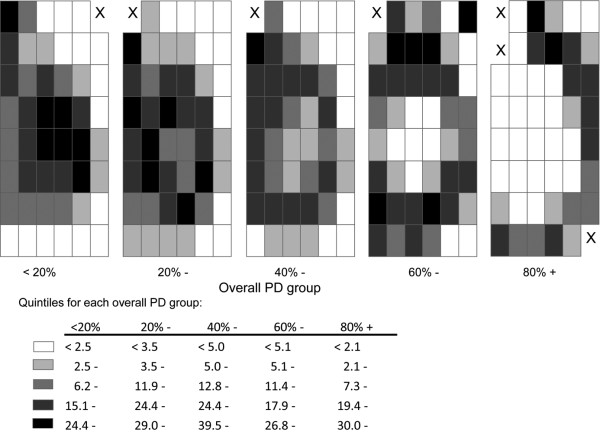
Between-woman variability in regional percent density, by level of overall percent density. For each region, between-woman variability in regional percent density (PD) was estimated by the standard deviation (SD) of the PDs for all participants in each overall PD group. X, region where fewer than two women contributed to the analysis and hence the SD could not be estimated.

Analyses stratified by the five predefined overall PD strata, and categorising the distribution of mean regional PDs within each one of these strata into quintiles, confirmed that the central regions were always the densest regardless of the value of the overall PD (Figure 2). There was, however, marked between-woman variability in regional PD as assessed by the SD of the distribution of PD for each region (Figure 3). For overall PD strata of < 20% and 20 to 39%, the between-individual variability in the regional PD was highest in the central regions and lowest along the skin edge. In contrast, for overall PD strata of 60 to 79% and ≥ 80%, the between-woman variability was lowest in the central regions and highest along the skin edge (Figure 3).

**Figure 4:**
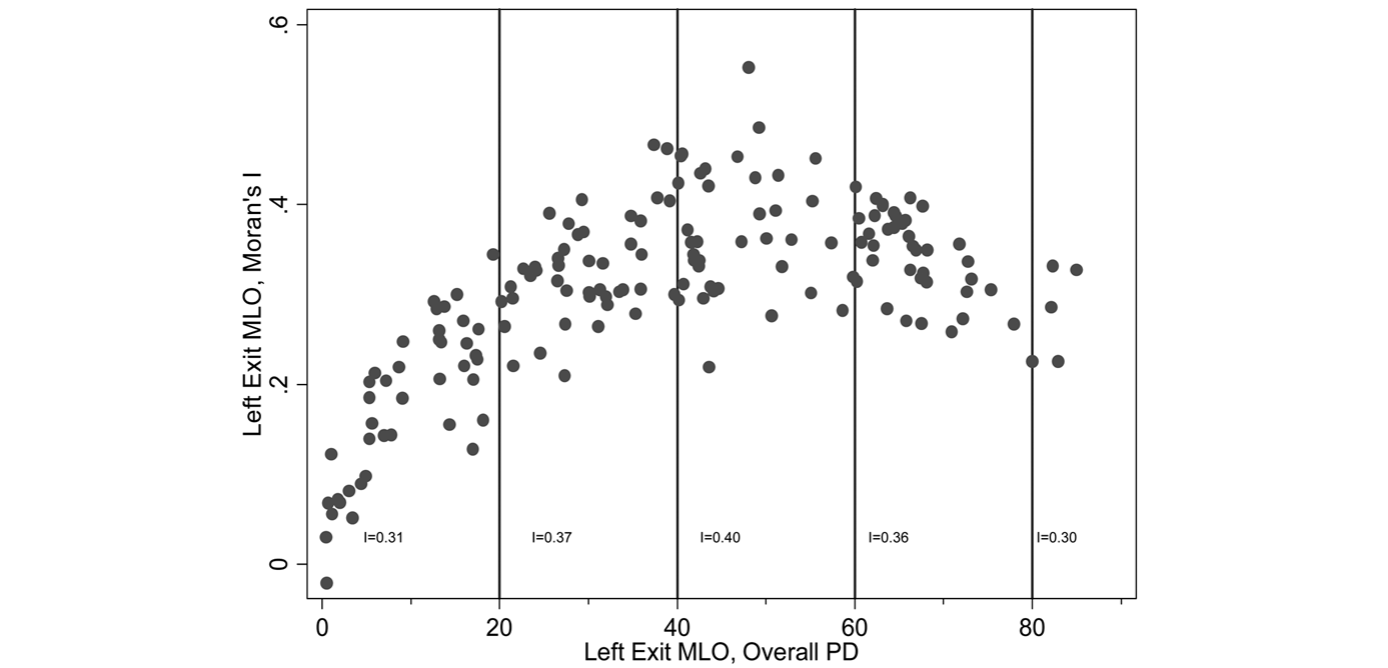
Moran's *I *value versus overall percent density. Moran's *I *value for each study participant plotted against her overall percent density (PD) (both at left exit medio-lateral oblique (MLO) screen). The Moran *I *values quoted on the bottom of the graph were calculated on a single averaged-image representing mean regional PDs for all participants in each overall PD group.

The median Moran *I *value for the 165 left exit MLO screens was 0.31 (interquartile range (IQR) = 0.26, 0.37), indicating a moderate degree of positive clustering on average. Image-specific *I *values, however, were related to the overall PD for the whole image (Figure 4). The *I *values increased progressively with increasing overall PD and were highest for overall PDs between 40% and 59% with a median of 0.36 (IQR = 0.31, 0.43), whereas for the least-dense breasts (< 20% overall PD) the median was 0.20 (IQR = 0.12, 0.25). For very low and very high overall PDs, most regions would have similar PD values and the whole breast would be relatively homogeneous in terms of its density; the curved relationship between the *I *value and the overall PD is therefore not surprising. Image-specific *I *values thus showed that density was clustered within a woman.

To assess whether the location of the low and high PD clusters differed between women, the *I *value for a single, averaged image representing the mean regional PD values for all 165 women combined was estimated as being 0.39; the *I *values for averaged images representing women in the < 20%, 20 to 39%, 40 to 59%, 60 to 79% and ≥ 80% overall PD strata were, respectively, 0.31, 0.37, 0.40, 0.36 and 0.30 (Figure 4). These single, averaged image *I *values indicated that the high and low PD clusters tend to be located roughly in the same regions of the breast across all women regardless of the level of their overall PD. Although these between-woman analyses were based on aggregated data and therefore interpretation might be affected by the ecological fallacy (that is, clustering may be present at an aggregated level but not at an individual level), their findings are consistent with the individual-based data shown in Figure 1b. The central regions broadly corresponded to the regions where, for most women, the regional PD was greater than the corresponding overall PD for the same breast and the same screen (Figure 1b).

**Figure 5:**
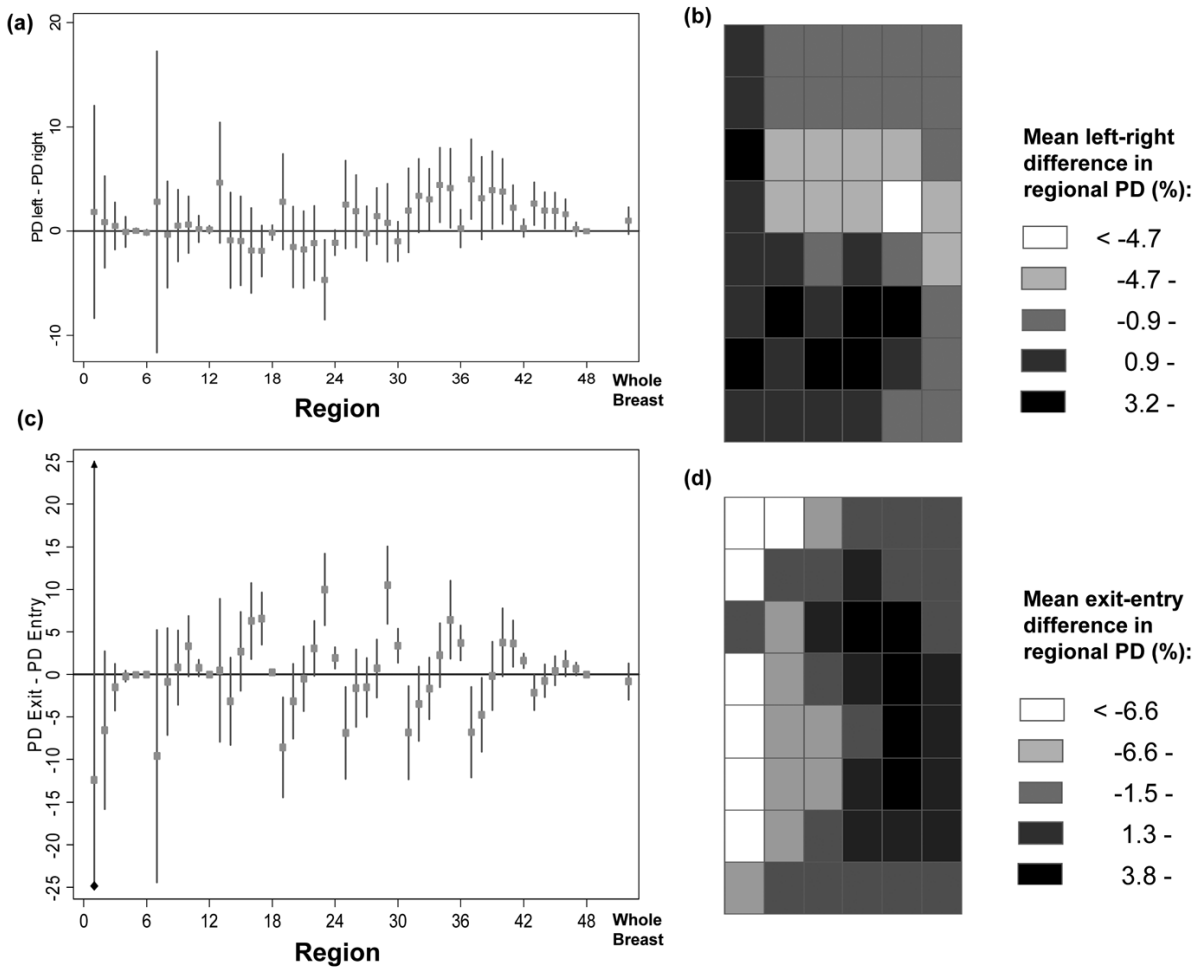
Left-right and exit-entry differences in regional percent density. Within-woman mean differences (with 95% confidence intervals) in percent density (PD) between equivalent regions **(a) **in the left and right breasts and **(c) **in the exit and entry screens, for each one of the 48 regions and for the whole breast. Spatial distribution of the within-woman mean differences **(b) **between the left and right breasts and **(d) **between the exit and entry screens in the regional PD.

Left – right breast comparisons were carried out on 164 women because the right exit MLO was missing for one participant. The spatial distribution of the mean regional PD, for all women combined, was similar between the left and right MLO screens (data not shown). The mean within-woman difference in overall PD between the left and right breasts was 0.99% (95% CI = -0.29%, 2.28%; *P *= 0.13). Mean within-woman differences in PD values between any two equivalent regions in the left and right breasts were rather small, ranging from -4.7% to 5.0% (median (IQR) = 0.6% (-0.2%, 2.6%)) (Figure 5a). Regions in the lower half of the left breast tended to have higher PD values than their counterparts in the right breast (Figure 5a, b), but these should be interpreted with caution because of differences in breast positioning and compression between the left and right screens. The mean within-woman difference in Moran's *I *values between the left and the right breasts was 0.00 (95% CI = -0.01, 0.01; *P *= 0.76; 95% limits of agreement (that is, mean difference ± 2 SDs) = -0.12, 0.12), indicating that the degree of density clustering was similar for the two breasts.

Neither parity nor age at fist birth (among parous women) affected the degree of spatial autocorrelation of density (adjusting for overall PD group and accounting for clustering within women). In contrast, as the BMI increased, the level of clustering of dense and nondense areas decreased (Table 2).

**Table 2:** Determinants of spatial autocorrelation in density

Number of women	Number of observations	Correlate of density	Categories	Change in Moran's *I *value from baseline^a^	*P *value^b^
165	493	Parity	0	Baseline	0.14
			1	0.04 (0.00, 0.07)	
			2	0.01 (-0.01, 0.04)	
			3+	0.03 (-0.00, 0.06)	
162^c^	484	Body mass index (kg/m^2^)	≤ 21.5	Baseline	0.03
			> 21.5 to ≤ 24.1	-0.02(-0.04, 0.00)	
			> 24.1 to ≤ 27.0	-0.01(-0.03, 0.01)	
			> 27.0	-0.04(-0.07, -0.01)	
138^d^	413	Age at first birth (years)	≤ 23	Baseline	0.25
			> 23 to ≤ 27	0.01 (-0.02, 0.03)	
			> 27 to ≤ 30	0.01 (-0.01, 0.04)	
			> 30	-0.01(-0.04, 0.01)	

^a^Change (95% confidence interval) estimated by regressing Moran's *I *value onto correlates of density while adjusting for overall breast density and clustering at the woman level. ^b^Wald's test. ^c^Body mass index missing for three women. ^d^Among parous women only.

The mean within-woman difference in overall PD between entry and exit screens taken an average of 8 years apart (Table 1) was 0.84% (95% CI = -1.30%, 2.97%; *P *= 0.44). The distribution of mean regional PD values, using aggregated data for all 164 women combined, remained relatively constant between the two screens (data not shown). Examination of within-woman differences in regional PD between the entry and the exit screens, however, showed that the direction and magnitude of these varied between regions. Regional PD decreased between the two screens for regions located centrally, but increased slightly in regions located around the skin edge (Figure 5c, d). The change in PD in any given region therefore did not always equal the woman's mean overall PD change (Wald test, *P *< 0.01), but some of these within-woman regional differences in age-related changes in PD might be accounted for by differences in breast size (Table 1), compression and positioning between the two screens. Similar findings were observed when proportional rate changes in regional PD were considered rather than absolute differences. Despite these regional variations in PD changes, the mean within-woman difference in Moran's *I *value between the two screens was 0.01 (95% CI = -0.01, 0.02; *P *= 0.30; 95% limits of agreement = -0.15, 0.16), indicating that a woman's degree of spatial autocorrelation in density did not change as she aged (during her forties).

At the entry screen 164 women had 487 points of highest density (median (IQR) points per woman = 3 (2, 4)), and at the exit screen 165 women had 404 points of highest density (median (IQR) = 2 (1, 3)). At both time periods, approximately 62% (301/487 and 252/404 points, respectively) of these highest-density points occurred in central regions (that is, regions 20 to 22, 26 to 28 and 32 to 34; Figure 1a). Using data from the 164 women that had a mammogram at both time points, the mean distance moved by a point of highest density was 1.35 regions (95% CI = 1.20, 1.50), and this distance varied according to PD group at the exit screen (*P *< 0.01; Table 3). The proportion of points of highest density that lay in regions of highest density (defined by the top quartile of regional density for each woman) at the left exit MLO screen was 0.91 (95% CI = 0.87, 0.94) for all women, and this also varied by PD group (*P *< 0.01; Table 3). The mean (SD) proportional increase in threshold value between the overall PD and the highest-density point was 0.67 (0.45), implying that the highest threshold value was usually less than twice the overall threshold value.

**Table 3:** Location of the points of highest density within the breast, by level of overall PD

Overall PD value at left exit MLO screen	Number of women	Proportion of highest-density points located in highest-density regions^a^	Mean entry-exit change in the location of the point of highest density^b^
All	165	0.9 (0.9, 0.9)	1.3 (1.2, 1.5)
< 20%	41	1.0 (0.9, 1.0)	1.4 (1.1, 1.7)
20 to 39%	43	1.0 (0.9, 1.0)	1.0 (0.7, 1.3)
40 to 59%	38^c^	0.9 (0.8, 1.0)	1.3 (1.0, 1.6)
60 to 79%	38	0.8 (0.7, 0.9)	1.6 (1.3, 1.9)
≥ 80%	5	0.8 (0.3,1.2)	2.4 (1.7, 3.1)
*P *value^d^		< 0.01	< 0.01

^a^The highest-density regions in each woman were those in the top quarter of her distribution of regional percent density (PD) values at the left exit medio-lateral oblique (MLO) screen. ^b^Expressed as the mean number of regions between the points of highest density between the entry and exit left MLO screens (see Methods) and accounting for clustering within women. ^c^Only 37 women in the change in location analysis. ^d^Wald's test.

## Discussion

The present study demonstrated that the distribution of radiodense tissue within the breast is spatially autocorrelated, with high-density areas clustering in the central regions of the breast regardless of the average level of density across the whole breast. In general, the clusters of high density and low density tended to be located roughly in the same regions within a woman (that is, between her left and right breasts) and among different women.

One of the main strengths of this study was that it provided an opportunity to examine within-woman longitudinal changes in the spatial distribution of density, although restricted to a relative narrow (and young) age range. We found evidence of some regional variability in the change in PD between the entry and exit screens, with declines in PD being restricted to the central regions of the breast. Despite this regional variability in the change in PD, the within-breast degree of spatial autocorrelation remained relatively constant over time – perhaps because the changes in regional PD were of a small magnitude relative to their absolute values, and therefore they did not affect the general pattern of density distribution within the breast. Further longitudinal studies covering a much wider age span are needed to examine the full effect of aging on the pattern of density distribution.

As expected, over 90% of the points of highest density were located in the densest regions of the breast, but this proportion varied slightly between women according to the level of their overall PD. Within a woman the location of the point of highest density changed only slightly between her exit and entry screens, but once again the extent of this change varied according to the level of her overall PD.

The findings from this study are consistent with our current knowledge of the biology of mammographic density. The observation that high-density areas tend to cluster in the central regions of the breast is not surprising as these regions correspond to the location of the mammary gland lobules, which are radiologically denser than the surrounding fatty tissue. Similarly, the observed decline in the PD of the central regions of the breast between the entry and the exit screens is consistent with the involution of the lobules of the breast with aging, and hence with the decline in the amount of radiodense fibroglandular tissue within the central regions of the breast [15,16]. The high within-woman concordance between the left and right breasts in terms of average density and degree of density clustering implies that, whatever the biological mechanisms affecting density, the main driving influences are likely to affect the two breasts similarly rather than having breast-specific or even more localised effects. This would be consistent with data from twin studies [17] and segregation analysis [18] indicating that breast density is a heritable quantitative trait, and with the known influences of age, parity, body weight, and circulating levels of certain hormones/growth factors [19], all of which are likely to affect the radiodense tissue in a generalised way rather than in a localised way.

Average density for the whole breast declines with successive pregnancies and increasing BMI [20]. Remarkably, neither parity nor age at first birth among parous women affected the degree of spatial autocorrelation, but higher BMI was associated with a decline in the degree of clustering of high-density and low-density areas. This decline in the degree of clustering may reflect the fact that a higher BMI was associated with a larger breast and hence larger-sized regions, with these being subject to greater PD variability.

It is not known whether the distribution of radiodense tissue may affect breast cancer risk independently of the average level of density for the whole breast. The findings from this study showed that, although the general pattern of spatial distribution was similar across all women regardless of level of average density, there was considerable between-women variability in the distribution of regional PD (as illustrated in Figures 1b and 3). It would be informative to assess whether such between-woman variation in the spatial distribution of radiodense tissue contributes independently to risk prediction. A recent study demonstrated that breast texture features derived from mammograms predicted breast cancer risk to a similar magnitude as PD, but did not specifically investigate the spatial distribution of such texture features [21].

It is also unclear whether mammographic density is a general marker of susceptibility to breast cancer or a more localised one, with cancers arising within the densest areas in the breast. Two studies have attempted to address this issue, but they have reported opposing findings [4,5]. The first study found that almost all (21/22) tumours arose within dense tissue [4]. The second larger study, however, found no association between regional density and tumour location [5]. Examination in large series of breast cancer cases of the extent to which the tumour location distribution coincides with the prediagnostic density distribution would be worthwhile. Several studies have examined the distribution of tumours within the breast [6,22-26], although their definitions of what constitutes the central area of the breast are not always clear or consistent. None of these studies, however, attempted to relate the tumour distribution to the distribution of radiodense tissue.

To our knowledge the present study is the first to have examined the spatial distribution of radiodense tissue within the breast. The study benefited from a sample of premenopausal women with repeat screens from a younger age (40 to 41 years) and with highly reliable measurements of density. Regions within the breast were defined in a consistent way, although the same region might have captured different areas of the breast across films due to differences in the size and positioning of the breast. This limitation may be overcome in the future through the use of registration techniques that combine translation, rotation, scaling, skewing and more complex nonrigid transformations to provide a more accurate correspondence between regions on serial films [27,28].

Moran's *I *values are affected by the choice of neighbouring regions and weights used in their calculation. Preliminary plots showed that, in general, as the distance between regions increased, the correlation between their corresponding PDs decreased. Neighbouring regions were therefore defined on the basis of distance rather than adjacency. To assess the robustness of the findings we considered alternative types of distance-based weights (that is, 1/*d*, e-^-*d *^and ). Although different types of weights yielded different ranges for the *I *values, the findings and conclusions of the study remained unchanged.

The identification of the points of highest density was conducted using a predefined method and was carried out in two sittings by a single reader (SMPP). Finally, the findings of this study are based on an area-based model for breast density where a pixel is dichotomised as dense or nondense. Volumetric methods of density classification should in theory be more accurate models of the true breast volume as regions with the same area-based PD may have quite different volumes of fibroglandular tissue [29]. Although physical spatial clustering of dense tissue within the breast is plausible based on these results and on breast anatomy, inferences must be made with caution as mammograms are a two-dimensional projection of a three-dimensional entity. In particular, the point of highest density on a two-dimensional mammographic image might simply reflect the overlay of tissues from different lobes located along the thickness of the breast. The point of highest density, as we have defined it, however, may still be of use in relation to tumour location on mammograms.

Three-dimensional technologies such as magnetic resonance imaging and tomosynthesis will overcome some of these limitations, but these imaging modalities are expensive and thus are not routinely performed.

## Conclusions

The present study is the first to demonstrate that the distribution of radiodense tissue within the breast is spatially autocorrelated, with the high-density areas clustering in the central regions of the breast. Within-woman comparisons showed that the degree of clustering of the high-density and low-density areas was similar between her two breasts and was little affected by age (within the rather narrow age range examined), despite evidence that age-related changes in PD were more marked in the central regions. Reproductive factors did not affect the degree of spatial autocorrelation of density, but the degree of clustering of high-density and low-density areas decreased as the BMI increased.

## Abbreviations

BMI: body mass index; CI: confidence interval; IQR: interquartile range; MLO: medio-lateral oblique; PD: percent density; SD: standard deviation.

## Competing interests

The authors declare that they have no competing interests.

## Authors' contributions

SMPP, VAM and IdSS conceived and designed the study, contributed financial support, and collected the data. IdSS and SMM recruited the study subjects. SMPP and VAM analysed the data. All authors interpreted the data and wrote the manuscript.
